# Association between Systemic Lupus Erythematosus and Periodontitis: A Systematic Review and Meta-analysis

**DOI:** 10.3389/fimmu.2017.01295

**Published:** 2017-10-17

**Authors:** Zoe Rutter-Locher, Toby O. Smith, Ian Giles, Nidhi Sofat

**Affiliations:** ^1^Musculoskeletal Research Group, Institute of Infection and Immunity, St George’s University of London, London, United Kingdom; ^2^Faculty of Medicine and Health Sciences, University of East Anglia, Norwich, United Kingdom; ^3^Center for Rheumatology Research, Rayne Institute, University College London, London, United Kingdom

**Keywords:** systemic lupus erythematosus, autoimmune and inflammatory diseases, microorganisms, periodontitis, periodontal disease, meta-analysis

## Abstract

**Background:**

Systemic lupus erythematosus (SLE) is a chronic systemic inflammatory autoimmune disease, the etiology of which remains only partially characterized. Strong evidence implicates chronic infections in the development and chronicity of autoimmune conditions. Recently, an association has been demonstrated between periodontitis and rheumatoid arthritis. Such observations have led to the investigation of the possible role of periodontitis and oral dysbiosis in other systemic inflammatory conditions, including SLE. The aim of this study was to examine whether there is an association between SLE and periodontitis.

**Methods:**

MEDLINE *via* OVID, EMBASE *via* OVID, and PsycINFO *via* OVID databases were searched to identify eligible studies, screened by two independent authors and verified by a third. Studies comparing presence of periodontitis in SLE cases to controls without SLE were included. Data were extracted using a predefined table and papers were appraised using Down’s and Black tool. Mantel–Haenszel meta-analysis was performed using RevMan.

**Results:**

Eight case–control studies were included, with 487 SLE cases and a total of 1,383 participants. On meta-analysis of four studies, risk of periodontitis in SLE cases compared to controls was significantly greater with a risk ratio of 1.76 (95% CI 1.29–2.41, *p* = 0.0004). No statistical difference was found in individual measures of periodontitis, such as probing depth or clinical attachment loss, between SLE cases and controls.

**Conclusion:**

Our study found a statistically significant increased risk of periodontitis in patients with SLE compared to controls. This finding suggests a possible association between these two conditions. Larger longitudinal studies are needed to confirm this possible association.

## Introduction

Systemic lupus erythematosus (SLE) is a systemic, chronic inflammatory condition with diverse clinical manifestations, primarily affecting the joints, internal organs, and the skin (1).

The etiology of SLE is incompletely understood, but it is thought to occur in genetically primed individuals in whom the inflammatory response is triggered by an environmental stimulus. Immunosuppressant medications are the mainstay of treatment, but these are limited both in efficacy and by multiple side effects which lead to significant morbidity and mortality. Oral manifestations of SLE are common and typically take the form of painless oral ulcers that are frequently present during disease flares and are included in current SLE classification criteria (2).

Periodontitis is an infectious-inflammatory condition, affecting the periodontal ligament and alveolar bone (3). Most cases are due to the chronic accumulation of oral plaque which initiates inflammation, further bacterial colonization and tissue destruction. Gingivitis usually occurs first and can be reversed with oral hygiene methods. However, once the inflammation extends past the gums to the deeper tissues, the loss of periodontal attachment and bone causes progressive loosening of teeth, eventually leading to their loss (4). The “red complex” organisms, *Porphyromonas gingivalis, Tannerella forsythia*, and *Treponema denticola*, have a key role in the development of periodontitis (5, 6).

There is strong evidence that periodontitis, and specifically oral dysbiosis is associated with autoimmune inflammatory disease, principally rheumatoid arthritis (RA). A recent meta-analysis including 153,492 participants showed a significant association between periodontitis and rheumatoid arthritis (7). Two specific bacteria have been implicated in triggering the underlying inflammatory process, *P. gingivalis* (8, 9) and *Aggregatibacter actinomycetemcomitans* (10). Clinical trials are underway to investigate the effect of non-surgical treatment of periodontitis, such as oral hygiene and mechanical removal of plaque in RA.

There is increasing interest regarding the possible role of oral dysbiosis in the etiology of other autoimmune inflammatory conditions, including SLE. Further understanding of any potential association between periodontitis and SLE would expand current knowledge of the etiology of SLE and may lead to novel management strategies.

In this review, we hypothesized that there may be an association between SLE and periodontitis. To evaluate this hypothesis, we conducted a systematic review and meta-analysis of relevant publications.

## Materials and Methods

The protocol for the review was registered with PROSPERO (Registration number: CRD42016053490) an international register of systematic reviews (http://www.crd.york.ac.uk/PROSPERO/). We used the Preferred Reporting Items for Systematic Reviews and Meta-Analyses (PRISMA) 2009 checklist to report the review (11).

### Eligibility Criteria for Population

Participants in the studies needed to have a diagnosis of SLE based on internationally recognized criteria, ACR 1982/1997 revised classification criteria (12) or clinical diagnosis by a rheumatologist. Studies which included participants of all ages, gender, and disease severity were eligible.

### Eligibility Criteria for Study

To be included, studies needed to be observational studies of cross-sectional, case–control, or cohort design. Journal articles and conference proceedings were included. Review articles, case reports, animal model studies, and those with unavailable abstracts were excluded. Non-English language papers were excluded. There were no restrictions on date of publication or publication status.

### Eligibility Criteria for Outcome Measure

To be included, prevalence of periodontitis using standardized measures needed to be reported in both SLE population and non-SLE population.

### Search Strategy

MEDLINE *via* OVID, EMBASE *via* OVID, and PsycINFO *via* OVID databases were searched using the following terms: systemic lupus erythematosus, SLE, lupus erythematosus, systemic or lupus nephritis, lupus vasculitis and Periodont*, gum disease, gingivitis, tooth decay, oral health, dental health, oral plaque index (PI), probing pocket depth, bleeding on probing (BOP), and clinical attachment loss (CAL). In addition, Google Scholar was searched using the following term “Periodontitis and systemic lupus erythematosus.” The searches were re-run just before the final analyses on the 03/08/2017.

### Study Selection

The Titles and/or abstracts were screened by two independent authors (Zoe Rutter-locher and Toby O. Smith) to identify potential eligible studies. Final selection of studies was performed by two independent authors (Zoe Rutter-locher and Toby O. Smith) and verified by a third author (Nidhi Sofat) by reviewing the full text based on inclusion criteria above. Any disagreements were resolved by discussion.

### Data Extraction

A standardized, pre-piloted form was used to extract data from the included studies. Extracted information included: (1) study design including, publication journal and date, inclusion and exclusion criteria, diagnostic criteria of SLE, definition of periodontitis, (2) study participant demographics including % females, mean age, years of SLE disease, therapies, measure of severity of SLE, and (3) periodontal measures including prevalence of periodontitis, oral plaque index (PI), probing depth (PD), clinical attachment loss (CAL), and bleeding on probing (BOP). Two authors (Zoe Rutter-locher and Toby O. Smith) extracted the data independently, discrepancies were identified and resolved through discussion (with a third author, Nidhi Sofat, where necessary). Authors were contacted by email to obtain missing information and included when received.

### Quality Assessment

Risk of bias and quality assessment was reviewed using Downs and Black tool for non-randomized control trials (13). This 27-point tool assesses studies on five key sections: (1) study quality (10 points), (2) external validity (3 points), (3) study bias (7 points), (4) confounding and selection bias (6 points), and (5) Power of the study (1 point). Disagreements between the review authors over the risk of bias in particular studies were resolved by discussion, with involvement of a third review author where necessary.

### Data Analysis

As there was homogeneity in participants, study design and outcome measure on visual assessment of the data extraction table, a meta-analysis was performed. Primary outcome was to calculate relative risk of periodontitis in participants with SLE compared to participants without SLE. Secondary outcomes were to calculate relative risk or mean difference for measures of periodontal disease. These measures of periodontal disease included PD, oral PI, BOP, bleeding gingival index, and CAL.

Median and interquartile range were converted to mean (SD) to allow comparison of studies, under the assumption of normal distribution. A fixed effect meta-analysis was performed when the inconsistency value (*I*-squared) was ≤50% and Chi-squared equates *p* = 0.10 and a random-effect meta-analysis when *I*-squared was >50% and Chi-squared equates to *p* < 0.10. Risk ratio with 95% confidence intervals was calculated for the prevalence rates of periodontitis and the mean difference was calculated for continuous variables. Risk of bias was identified in Down’s and Black tool. Publication bias was not performed as it is convention to only present a funnel plot for 10 or more data points in a meta-analysis (14).

All analysis and forest plots were performed on RevMan Version 5.3 (Copenhagen: The Nordic Cochrane Centre, The Cochrane Collaboration, 2014).

## Results

### Search Results

**Figure 1:**
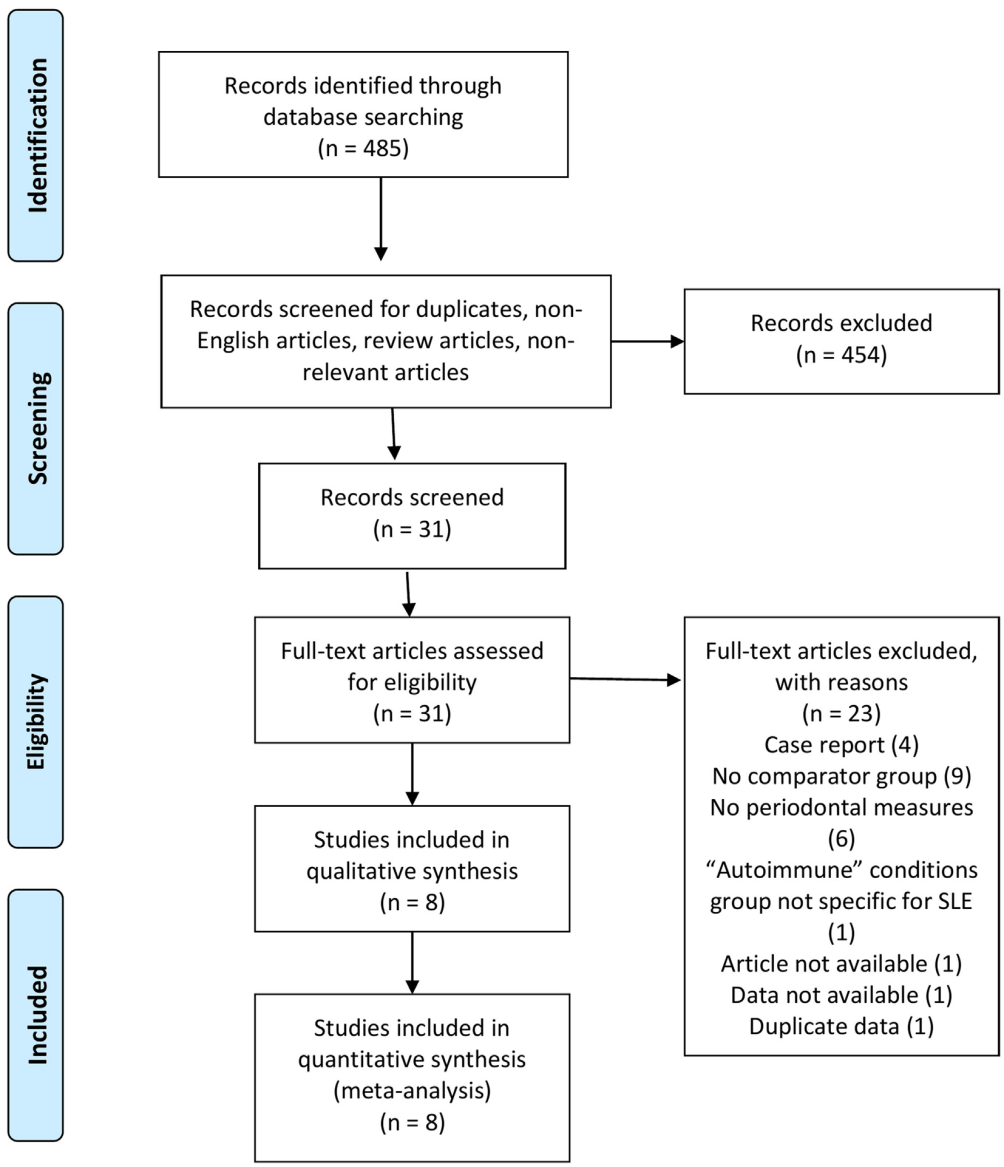
PRISMA flow diagram showing results of search strategy.

As shown in Figure 1, a total of 485 studies were identified using the search strategy. Of these, 454 were excluded as they were duplicates, non-English articles, review articles, or non-relevant. The 31 remaining full length articles were screened and eight were deemed appropriate to be included in the qualitative and quantitative analysis.

### Study Design

The study characteristics are shown in Table 1 and Table S1 in Supplementary Material. All studies included in the meta-analysis were case-control in design. Seven were published in peer review journals and one was taken from conference proceedings (15). Most studies (six of seven) were published since 2015. The remaining articles were published between 1993 and 2007. Three studies were based in Europe (two in the UK and one in Germany), two in Brazil, one in Saudi Arabia, one in Taiwan, and one in China.

**Table 1:** Study Characteristics.

Reference	Country	Time period	Systemic lupus erythematosus (SLE) cases	Control	Inclusion criteria cases	Exclusion criteria cases
Al-Mutairi et al. (16)	Saudi Arabia	2012–2014	25	50	Diagnosis SLE, female, >20 years old	Smoking, pregnancy, diabetes mellitus, history of periodontal treatment in the preceding 6 months, antibiotic prophylaxis
Calderaro et al. (17)	Brazil	2013–2014	75	75	Diagnosis SLE, >18 years old, >8 teeth	SLE overlap diseases, treatment of periodontitis in the preceding 6 months, antibiotics in the preceding 3 months, antibiotic prophylaxis, end-stage renal failure, pregnancy or lactation, neoplasia in preceding 5 years, acute or chronic infections
de Pablo et al. (15)	UK	Not stated	105	484	Diagnosis SLE	Unknown
Fernandes et al. (18)	Brazil	2004–2005	48	48	Diagnosis juvenile SLE	Current periodontal treatment
Meyer et al. (19)	Germany	1995–1996	46	50	Diagnosis SLE	Current antibiotic or antiviral treatment
Mutlu et al. (20)	UK	Not stated	27	25	Diagnosis SLE	Pregnancy, antibiotics in the preceding 6 months, history of periodontal disease
Wang et al. (21)	Taiwan	2012–2013	53	56	Diagnosis SLE	Smoking, antibiotics in the preceding 3 months, periodontal treatment in the preceding 3 months
Zhang et al. (22)	China	2015–2016	108	108	Diagnosis of SLE	Hearing and cognitive impairment, diabetes, renal failure, pregnancy, and lactation, cancer, history of periodontal treatment preceding 3 months, antibiotics preceding 3 months, number teeth <4

The total number of study participants was 1,383 which included 487 cases of SLE. All studies defined cases as fulfilling the ACR 1982/1997 revised classification criteria for diagnosis of SLE (12). Cases were recruited mainly from Rheumatology clinics, with the exception of Meyer et al. who recruited from the “Department of Internal Medicine” (19). All studies defined controls as either “Healthy” or “Individuals without a history of rheumatic conditions or autoimmune disease.” Controls were recruited from a variety of sources including dental clinics, staff at the medical school and epidemiological survey. Most studies included exclusion criteria in order to reduce the effect of confounders such as age, smoking, recent antibiotics usage, but this varied between studies.

### Quality Assessment

Quality assessment was hindered by the limited information available for some studies, especially those that came from conference proceedings. As shown in Table 2, studies included in this meta-analysis had clear hypothesis, outcome measures and aims (*N* = 8, 100%). The participants were representative of the population (*N* = 7, 88%) and the outcome measures were reliable and clearly reported (*N* = 8, 100%) with estimates of random variability (*N* = 7, 88%). Particular limitations were that assessors were not blinded (*N* = 1, 13%) and sufficiently powered cohort size was present in only half of studies (*N* = 4, 50%).

**Table 2:** Down and Black’s appraisal.

	1	2	3	4	5	6	7	8
Hypotheses/aims/objectives clearly described	✓	✓	✓	✓	✓	✓	✓	✓
Main outcome measures clearly described	✓	✓	✓	✓	✓	✓	✓	✓
Characteristics of patients/subjects clearly described	χ	✓	NS	✓	χ	χ	✓	✓
Interventions of interest clearly described	✓	✓	✓	✓	✓	✓	✓	✓
Distribution of principal confounders in each group clearly described	✓	✓	NS	✓	χ	χ	✓	✓
Main findings clearly described	✓	✓	✓	✓	✓	✓	✓	✓
Estimates of random variability in the data provided	✓	✓	✓	✓	✓	✓	χ	✓
Important adverse events reported	N/A	N/A	N/A	N/A	N/A	N/A	N/A	N/A
Characteristics of patients lost to follow-up described	N/A	N/A	N/A	N/A	N/A	N/A	N/A	N/A
Actual probability values reported	✓	✓	✓	✓	✓	✓	✓	✓
Participants approached representative of entire population	✓	✓	✓	χ	✓	✓	✓	✓
Participants recruited representative of entire population	✓	✓	✓	χ	✓	✓	✓	✓
Staff, places, and facilities were patients treated representative of majority of population	✓	✓	✓	χ	✓	✓	✓	✓
Blinding of study subjects	N/A	N/A	N/A	N/A	N/A	N/A	N/A	N/A
Blinding of assessors	χ	χ	NS	χ	χ	χ	χ	✓
Data based on data-dredging clearly stated	N/A	N/A	N/A	N/A	N/A	N/A	N/A	N/A
Time period between the intervention and outcome the same for cases and controls	N/A	N/A	N/A	N/A	N/A	N/A	N/A	N/A
Appropriate statistical tests used	✓	✓	NS	✓	✓	✓	✓	✓
Compliance to intervention reliable	N/A	N/A	N/A	N/A	N/A	N/A	N/A	N/A
Main outcome measure reliable and valid	✓	✓	✓	✓	✓	✓	✓	✓
Intervention groups or case–controls recruited from same population	✓	✓	✓	✓	NS	χ	NS	✓
Intervention groups or case–controls recruited at the same time	✓	✓	NS	✓	✓	✓	✓	✓
Study subjects randomized to the interventions	N/A	N/A	N/A	N/A	N/A	N/A	N/A	N/A
Was concealed randomization to allocation undertaken	N/A	N/A	N/A	N/A	N/A	N/A	N/A	N/A
Adequate adjustment made in the analysis of confounders	✓	✓	✓	✓	χ	✓	✓	✓
Patient losses accounted for	N/A	N/A	N/A	N/A	N/A	N/A	N/A	N/A
Sufficiently powered cohort size	χ	✓	✓	χ	χ	χ	✓	✓

*S, not stated; N/A, not applicable*.

*1, Al-Mutari et al. (16); 2, Calderaro et al. (17); 3, de Pablo et al. (15); 4, Fernandes et al. (18); 5, Meyer et al. (19); 6, Mutlu et al. (20); 7, Wang et al. (21); 8, Zhang et al (22)*.

### Study Participant Demographics

Table 3 shows the demographic of the cohorts. A total of 1,383 participants were included in the meta-analysis, with 487 participants with SLE and 896 participants without SLE. Mean age of participants with SLE was 41.3 years and controls was 42.8 years, excluding juvenile SLE. All studies had a predominance of females. Mean duration of disease was, as expected, lowest in study by Fernandes et al. looking at juvenile SLE (18). Excluding this study, mean disease duration ranged from 4.5 to 11 years. Use of immunosuppressant’s varied from 36 to 100%. There was also variability in what medications were included under the term “immunosuppression.” There was insufficient detailed data in order to control for immunosuppressant use in meta-analysis. Importantly, smoking status, a known risk factor for periodontitis, was the same in cases and controls in those studies with available data.

**Table 3:** Demographic of cohorts.

Reference	Mean age SLE/controls	% Females, SLE/controls	Years of SLE disease (mean)	Use prednisolone (% SLE)	Use of immunosuppressant (% SLE)	Smoking status SLE/controls
Al-Mutairi et al. (16)	33/37	100/100	6.5	100	36	0/0
Calderaro et al. (17)	38/41	91/77	11	83	79	11/11
de Pablo et al. (15)	46/49	92/56	DM	DM	DM	DM
Fernandes et al. (18)	14/13	85/67	3.1	98	48	0/0
Meyer et al. (19)	40/46	DM	4.5	DM	DM	DM
Mutlu et al. (20)	48/44	96/92	DM	81^a^	81^a^	DM
Wang et al. (21)	47/44	100/100	DM	DM	100	0/0
Zhang et al. (22)	37/39	100/100	7.5	67	41	DM

*DM, data missing*.

*^a^Use prednisolone or immunosuppression*.

### Meta-analysis: SLE and Periodontitis Prevalence

As shown in Table 4, all studies used similar measures of periodontitis. Four of the seven studies, which included 1,064 participants, defined periodontitis (15, 17, 21–23). Three studies (15, 17, 22) differentiated between mild and severe periodontitis.

**Table 4:** Definition of periodontitis and measures of periodontitis.

Reference	Definition of periodontitis	Measure oral plaque build up	Measure gingivitis	Measure periodontitis
Al-Mutairi et al. (16)	None	PI	BOP	RT, PD, CAL
Calderaro et al. (17)	≥2 sites PD ≥ 4 mm and ≥2 sites CAL ≥ 3 mm or 1 site PD ≥ 5 mm	PI	BOP	PD, CAL
de Pablo et al. (15)	≥1 sites PD ≥ 5 mm	None	None	PD
Fernandes et al. (18)	None	PI	BOP	DMFT
Meyer et al. (19)	None	PI	BOP	Bone loss, RT, DMFT
Mutlu et al. (20)	None	Pl	Loe and Silness GI	RT, PD
Wang et al. (21)	≥20% of tooth sites with PD ≥ 4 mm or CAL ≥ 4 mm	None	None	TT, PD, CAL
Zhang et al. (22)	CAL > 3 combined with panoramic radiographs	PI	BOP, GI	Bone loss, PD, CAL

*PI, oral plaque index; BOP, bleeding on probing; GI, gingival index; MT, residual teeth; PD, probing depth; CAP, clinical attachment loss; DMFT, decayed-missing-filled index; CAL, clinical attachment loss*.

**Figure 2:**
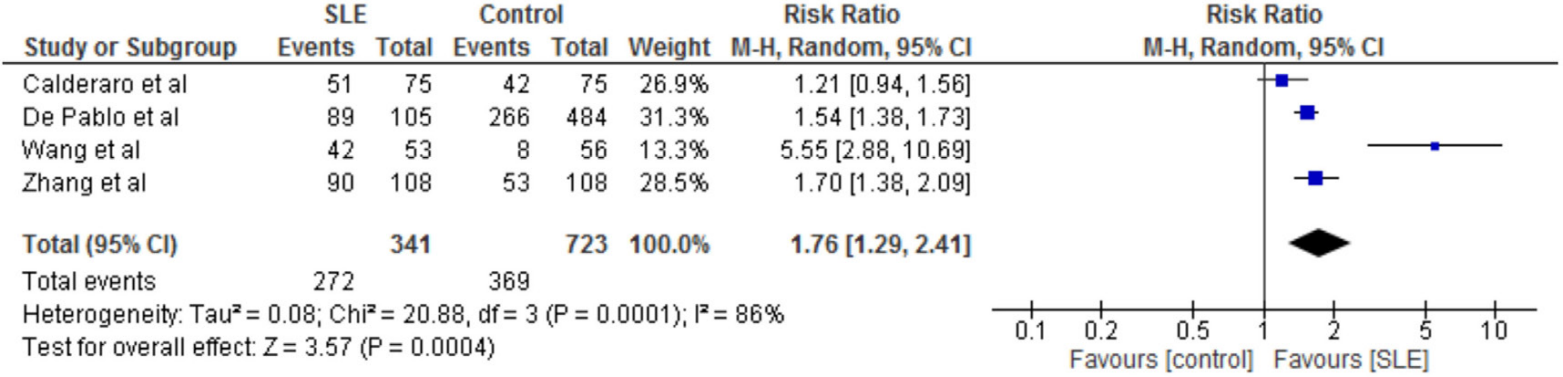
Forest-plot representing risk ratio of periodontitis between cases with systemic lupus erythematosus (SLE) and healthy controls.

The prevalence rates of periodontitis were similar in these four studies. The exceptions were the lower prevalence rate of periodontitis in the controls of the study by Wang et al. and Zhang et al. (21, 22). On meta-analysis, risk of periodontitis in SLE patients compared to controls was significantly greater with a risk ratio of 1.76 (95% CI 1.29–2.41, *p* = 0.0004, Figure 2). Furthermore, this greater risk ratio remained significant when the study published as a conference proceeding was excluded from the meta-analysis (RR 2.05 95% CI 1.15–3.66, *p* = 0.02) and when the study by Wang et al. was excluded from meta-analysis (RR 1.50 95% CI 1.28–1.75, *p* < 0.00001). Interestingly two studies which differentiated between mild and severe periodontitis found no difference in the prevalence of severe periodontitis between cases and controls, whereas one did (22).

### Meta-analysis: SLE and Measures of Periodontal Disease

All studies provided data on measures of periodontal disease. Six of the eight studies reported oral PI (16, 18–20, 22, 23). Six of the studies reported on presence of gingivitis (16–20, 22, 23), with five reporting BOP and two Loe and Silness gingival index (20, 22). Six studies provided data on PD (15–17, 20–23) and four on CAL (16, 17, 21–23) which are the gold standard measures of periodontitis. Four studies reported on residual teeth (16, 19–21), and two reported the WHO DMFT index (decayed-missing-filled teeth index) (18, 19). Meyer et al. and Zhang et al. were the only studies to use radiographs to measure bone loss, an accurate measure of periodontitis (19, 22).

Number of residual teeth was very similar in all studies. Overall, the mean residual teeth count was 23.68 for SLE and 23.65 for controls. On meta-analysis there was no significant difference in mean of PI (0.03, 95% CI −0.09–0.16, *p* = 0.62, Table 5, Figure S1 in Supplementary Material) or BOP (1.60, 95% CI −0.72–3.92, *p* = 0.18, Table 5, Figure S2 in Supplementary Material).

**Table 5:** Results from meta-analysis.

Outcome	Relative risk (95% CI)	*p* value	*N*	Statistical heterogeneity (*I*^2^; Chi^2^)
Periodontitis	1.76 (1.29–2.41)	<0.01	1,064	86%; 0.0001
Pl^a^	0.03 (−0.09–0.16)	0.62	685	94%; <0.0001
Bleeding on probing^a^	1.60 (−0.72–3.92)	0.18	633	41%; 0.15
Probing depth (PD) ≥ 5 mm	1.34 (0.99–1.82)	0.06	923	68%; 0.03
Clinical attachment loss (CAL) ≥ 2 mm	1.09 (0.99–1.21)	0.08	334	42%; 0.18
PD (mm)^a^	0.08 (−0.27–0.43)	0.66	493	99%; <0.0001
CAL (mm)^a^	0.41 (−0.12–0.95)	0.13	441	87%; 0.0005

*^a^Mean difference analysis*.

*CI, confidence intervals*.

We also performed meta-analysis on the gold standard measures of periodontitis. Risk of PD ≥ 5 mm was greater in SLE patients compared to controls but this was not statistically significant with risk ratio of 1.34 (95% CI 0.99–1.82, *p* = 0.06, Table 5, Figure S3 in Supplementary Material). Risk of CAL ≥ 2 and difference in means for PD and CAL were not statistically different between cases and controls; Risk of CAL ≥ 2 risk ratio 1.09 (95% CI 0.99–1.21, *p* = 0.08, Table 5, Figure S4 in Supplementary Material), PD difference in means 0.08 (95% CI −0.27–0.43, *p* = 0.66, Table 5, Figure S5 in Supplementary Material), CAL difference in means 0.41 (95% CI −0.12–0.95, *p* = 0.13, Table 5, Figure S6 in Supplementary Material). Excluding the study in Juvenile SLE by Fernandes et al. did not make a significant difference to any outcome.

## Discussion

Our report is the first systematic review to examine the association between periodontitis and SLE. On meta-analysis we found a statistically significant overall increased risk of periodontitis in patients with SLE compared to controls, suggesting an association between these two conditions. However, there was no statistical difference in individual measures of periodontitis, such as PD or CAL, between SLE cases and controls.

We found no significant difference in oral PI or BOP between SLE cases and controls. Plaque induced periodontitis is the most common form of periodontitis, and so a high oral PI would suggest a greater risk of developing periodontitis. BOP is a measure of gingivitis, the reversible gingival inflammation which precedes periodontitis. Therefore, although these measures do highlight potential risk of periodontitis, the absence of a significant difference between cases and controls does not preclude an association between SLE and periodontitis.

There was also no difference in means of PD. PD calculates the depth of periodontal pockets and is a measure of current periodontal disease. The British Society of Periodontology delineates a healthy sulcus as <3.5 mm and a periodontal pocket as ≥3.5 mm (24). We propose that calculating differences in mean PD < 3.5 mm is, therefore, not clinically significant and risk ratio of PD above a certain level is more appropriate. In this case, the finding that PD ≥ 5 mm was higher in SLE potentially supports the evidence that periodontitis is associated with SLE. However, as this was not significant further studies with larger sample size will be needed to elucidate this further.

Clinical attachment loss is the other gold standard measurement for periodontitis (25) and is representative of cumulative destruction. Although CAL ≥ 2 mm and mean difference in CAL was higher in SLE compared to controls, this not significantly different. Again, further studies are needed to investigate this relationship.

The finding that periodontitis is associated with SLE is in agreement with other studies examining this relationship. Case studies since the 1980s have suggested a link between SLE and gum disease. Rhodus and Johnson found 93.8% of SLE patients had periodontitis (26), while a Japanese study reported a prevalence of periodontitis of 70% in SLE compared to 30% in the general population (27). Higher disease activity, measured by SLE Disease Activity Index (SLEDAI) (28), predicts worse periodontal disease (24, 29) and non-surgical treatment of periodontitis improves SLEDAI scores at 3 months (30). These findings suggest that oral dysbiosis may contribute to maintenance of the inflammatory process in SLE. Recently, differences in the composition of the oral microbiota, independent of periodontal status, have been elucidated using 16s ribosome sequencing in 52 cases of SLE (23). Interestingly, the periodontal pathogen *Aggregatibacter actinomycetemcomitans*, which has been identified as a potential trigger in RA (10), has also been implicated in SLE (31).

Systemic lupus erythematosus is thought to occur when an environmental stimulus triggers inflammation in a genetically primed individual. Initial studies have highlighted possible mechanisms to explain the potential association between periodontitis and SLE. Genetic variants in the Fcy receptor have been implicated in susceptibility to both SLE and periodontitis in a small Japanese study (32). Periodontitis and SLE are both inflammatory conditions, and share similar inflammatory profiles (33, 34). Specifically, a possible role for TLR-4 has been implicated. These molecules are activated by specific pathogen-associated molecular patterns produced by bacteria and stimulation of TLR-4 leads to autoimmune lupus in mice (35). However, it must be emphasized that these studies are small and much more work is needed to elucidate if there is any biological plausibility.

### Study Limitations

A significant limitation to our meta-analysis is the lack of clear and recognized criteria to define periodontitis. Although all definitions of periodontitis used PD and CAL, they did vary in their thresholds, limiting the ability to directly compare outcomes. However, a minimum diagnostic threshold of CAL ≥ 2 mm and PD ≥ 3 mm has been suggested (25) and all studies used thresholds which surpassed these cutoffs.

Another significant limitation is the paucity of data available. Only eight studies could be included in the meta-analysis. The overall periodontitis risk ratio included only four studies, and one of these was from conference proceedings. Meta-analysis involving greater number of studies analyzing individual measures of periodontitis were not significant. This may suggest that the significant risk ratio in overall periodontitis cannot be extrapolated into larger study populations. However, previous similar meta-analysis in other conditions, which have included larger numbers of studies, have also found that overall periodontitis risk is significant, while individual markers are not (7).

There are only a small number of studies to date investigating this association and so we were unable to test for publication bias. We included data from conference proceedings try to mitigate this but it will be important in the future to ascertain the risk of small sample size publication bias.

There are a number of factors such as smoking, educational level, and immunosuppressant medications which increased risk of both SLE and periodontitis (1). Variation in the prevalence of these factors between cases and controls could, in part, be responsible for the differences seen. These factors were partly controlled for by the use of exclusion criteria in some studies. However, there was limited detailed information regarding smoking status and immunosuppressant use in most studies, and information that was available showed variations in immunosuppressant use from 36 to 100%.

Finally, all studies included were cross-sectional in nature and investigated an association at a given time point. We are, therefore, unable to make any conclusions regarding causality from this study. Further longitudinal studies are needed to delineate a temporal association and causality of periodontitis in the development of SLE.

## Conclusion

The results of this meta-analysis show a significant association between SLE and periodontitis. However, the meta-analysis was hampered by paucity of data and significant limitations. Therefore, these findings can only suggest a possible association and larger longitudinal studies are needed to confirm this association and investigate causality of periodontitis in SLE.

## Author Contributions

ZR-l, TS, and NS conceived, analyzed, and drafted the manuscript. IG conducted literature searches and reviewed the manuscript. All authors approved the manuscript for publication.

## Conflict of Interest Statement

The authors declare that the research was conducted in the absence of any commercial or financial relationships that could be construed as a potential conflict of interest.
