# Supplementary material for: Association between Systemic Lupus Erythematosus and Periodontitis: A Systematic Review and Meta-analysis
Source: Front Immunol. 2017 Oct 17;8:1295. doi: 10.3389/fimmu.2017.01295 (PMC5650969; doi:10.3389/fimmu.2017.01295)

Supplementary Material

**Association between Systemic Lupus Erythematosus and Periodontitis: A Systematic review and Meta-analysis**

Zoe Rutter-locher^1^, Toby O. Smith^2^, Ian Giles^3^, Nidhi Sofat^1^ *

^1^Musculoskeletal Research Group, Institute of Infection and Immunity, St George’s University of London, London, UK

^2^Faculty of Medicine and Health Sciences, University of East Anglia, Norwich, UK

^3^ Center for Rheumatology Research, Rayne Institute, University College London, London, UK

^*^**Correspondence:**

Dr Nidhi Sofat

[n.sofat@sgul.ac.uk](mailto:n.sofat@sgul.ac.uk)

Supplementary tables:

**Supplementary table 1: Additional information on Study Characteristics**

| **First author** | **Publication date** | **Journal** | **Recruitment cases** | **Recruitment controls** | **Inclusion criteria Control** | **Exclusion criteria control** |
| --- | --- | --- | --- | --- | --- | --- |
| Al-Mutari et al (16) | 2015 | Saudi Medical Journal | Rheumatology clinic | Dental clinic | Individuals without history of SLE or autoimmune conditions, Female,>20 years old | Same as case |
| Calderaro et al (17) | 2015 | Clinical Rheumatology | Rheumatology clinic | Domiciliary visits performed by AHPs | Individuals without rheumatic disease, Matched for age, educational level, monthly income | Same as case |
| De Pablo et al (15) | 2015 | EULAR conference proceeding | Unknown | Adult dental health survey | Individuals in Adult dental health survey, a representative population survey, Geographically matched | Unknown |
| Fernandes et al (18) | 2007 | Lupus | Paediatric Rheumatology clinic | Dental clinic | Healthy individuals, Matched for age | Same as case |
| Meyer et al (19) | 1999 | Journal of Oral Pathology and Medicine | Department of Internal medicine | Unknown | Healthy individuals, Matched for age and gender | Same as case |
| Mutlu et al (20) | 1993 | Community Dental Health | Rheumatology clinic | Staff/students medical school | Healthy individuals, matched for age and gender | Same as case |
| Wang et al (21) | 2015 | Journal of Periodontology | Rheumatology clinic | Unknown | Healthy individuals, matched for age, gender, race | Same as case |
| Zhang et al (22) | 2017 | Rheumatology International | Rheumatology clinic | Medical centre | Healthy individuals, matched for age and gender | Same as case |

Supplementary figures:

**Figure 1: Forest-plot representing difference in means of PI between cases with SLE and healthy controls.**


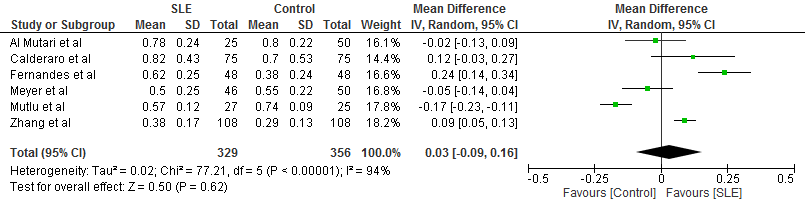


**Figure 2: Forest-plot representing difference in means of BOP between cases with SLE and healthy controls.**


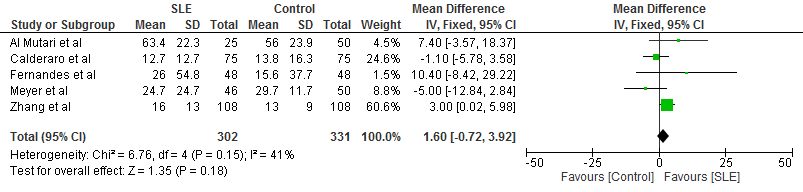


**Figure 3: Forest-plot representing risk ratio of PD≥5mm between cases with SLE and healthy controls.**


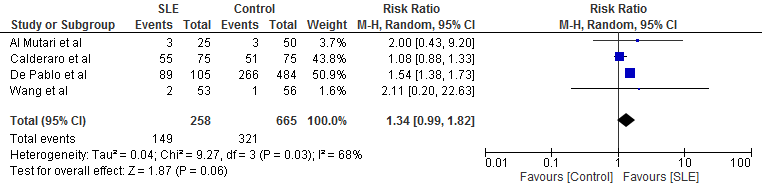


**Figure 4: Forest-plot representing risk ratio of CAL ≥2 between cases with SLE and healthy controls.**


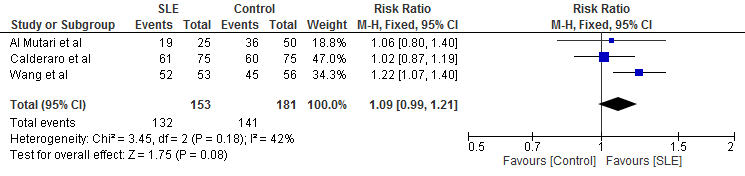


**Figure 5: Forest-plot representing difference in means of PD between cases with SLE and healthy controls**


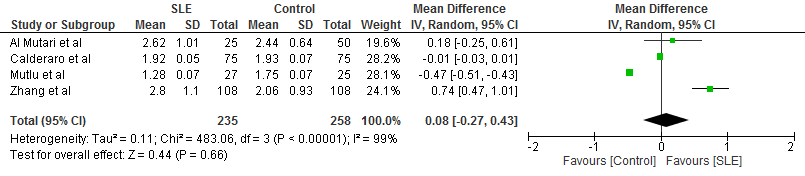


**Figure 6: Forest plot representing difference in means of CAL between cases with SLE and healthy controls.**


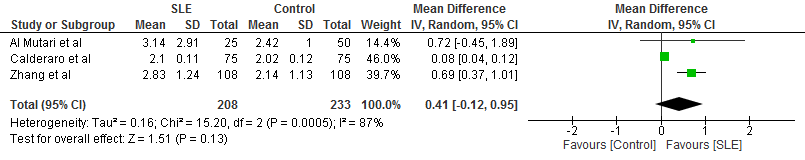

Supplement: Supplementary file 1 [file Table_1.DOCX]
